# Validity of the EASYCare Standard 2010 assessment instrument for self-assessment of health, independence, and well-being of older people living at home in Poland

**DOI:** 10.1007/s10433-017-0422-7

**Published:** 2017-04-03

**Authors:** Sławomir Tobis, Krystyna Jaracz, Dorota Talarska, Sylwia Kropińska, Ewa Zasadzka, Mariola Pawlaczyk, Katarzyna Wieczorowska-Tobis, Ian Philp, Aleksandra Suwalska

**Affiliations:** 1 Department of Geriatric Medicine and Gerontology, University of Medical Sciences, Poznan, Poland; 2 Department of Neurological and Psychiatric Nursing, University of Medical Sciences, Poznan, Poland; 3 Department of Preventive Medicine, University of Medical Sciences, Poznan, Poland; 4 Laboratory of Geriatrics, Department of Palliative Care, University of Medical Sciences, Poznan, Poland; 5 Warwick Business School, University of Warwick, Coventry, UK; 6 Laboratory of Neuropsychobiology, Department of Psychiatry, University of Medical Sciences, Poznan, Poland

**Keywords:** EASYCare, Self-assessment, Older people, Independence, Functional status

## Abstract

EASYCare Standard 2010 is a brief instrument identifying concerns in health, functional independence, and well-being, from older persons’ perspective. It has not previously been validated for self-assessment. Our aim was to determine whether self-assessment (EC1) can give comparable results to an evaluation performed by professionals (EC2), for older people living at home. The study included community-dwelling individuals (aged at least 60 years, *n* = 100; 67 females) without dementia (abbreviated mental test score [AMTS] above 6). It comprised two assessments (self and professional), including summarising indexes: *Independence score [IS], Risk of breakdown in care [RBC], Risk of falls [RF],* performed within a period between 1 and 2 weeks. Additionally, during EC1, reference tests of physical and mental function (Barthel Index: 96.3 ± 6.5, Lawton scale: 6.7 ± 2.0, geriatric depression scale: 3.0 ± 2.7, AMTS: 10.2 ± 1.0) were applied to test for concurrent validity. Cohen’s kappa values (self-assessment vs. professional assessment) across all EASYCare domains were high (0.89–0.95). Results of all summarising indexes derived from self-assessment correlated strongly with reference tests. No differences were found in IS and RBC between EC1 and EC2 (8.6 ± 12.0 vs. 9.0 ± 12.7 and 1.0 ± 1.1 vs. 1.2 ± 1.4). Results of RF were higher in EC2 (1.0 ± 1.1 vs. 1.1 ± 1.4; *p* = 0.005), due to a different response to the item “Do you feel safe outside your home?” We conclude that self-assessment with EASYCare Standard in older people without severe functional impairment living at home can deliver valid results, similar to those obtained through professional assessment, thus providing an efficient system for assessment of relatively independent individuals.

## Introduction

The number of older people is increasing rapidly on a global scale, with impact on the needs of services arising from functional limitations. There is growing interest in person-centred care which responds to individual needs. The designation is “person-centred” rather than “patient-centred”, as it involves more than just the health care (American Geriatrics Society Expert Panel on Person-Centered Care 2016). This concept of care provides an adequate support for independence which, in turn, both prevents functional decline and improves well-being (van der Bij et al. 2002).

Comprehensive geriatric assessment (CGA) is a multidisciplinary diagnostic process which is believed to be the best method to integrate the care for frail older people (Leichsenring 2004), to plan treatments, and to monitor outcomes. As it is a very complex procedure, a simpler method is needed for everyday use in preventive care for older people living in the community. It should cover a variety of aspects of social and health domains in order to screen for individuals who are in need of support and have increased risk of dependence. It should also incorporate established assessment methods where possible (Philp 1997). The questionnaire EASYCare Standard 2010 may constitute such an instrument (Philip et al. 2014).

The EASYCare questionnaire was introduced in 1997 and subsequently translated into many languages, including Polish (Bień et al. 1999), with reliability and validity demonstrated in various countries in all World Health Organisation (WHO) regions (Philip et al. 2014). In 2010, the tool was presented as EASYCare Standard 2010—an extended version that benefited from research and clinical activities performed to that date, introducing three summarising indexes *(Independence score, Risk of breakdown in care, Risk of falls)* which briefly characterise the subject’s status (Pınar et al. 2015; Jotheeswaran et al. 2016; Brandão et al. 2015).

It incorporates elements of basic activities of daily living (ADL), selected instrumental activities of daily living (IADL, e.g. cooking, shopping, paying the bills, travelling, etc.), items on safety, accommodation and finances from WHO multinational survey (WHO 1983), and specific items on well-being (Olde-Rikkert et al. 2013). It was shown to be an effective instrument when administered by both health care (van Eijken et al. 2008) and social care practitioners (Clarkson et al. 2009). The use of the instrument by general practitioners (GPs) was effective in prediction of negative health outcomes within one year from the assessment (van Kempen et al. 2015). Also its cost-effectiveness was shown in the comparison with usual care in frail older people at 6-month follow-up (Melis et al. 2008). The results of our previous study in a group of older subjects at an oncological surgery clinic (Talarska et al. 2016) showed that the use of the questionnaire allowed to identify functional limitations of older people for planning individual support in order to minimise the risk of disability and hospitalisation. Correlation was found between EASYCare results and those obtained with ADL and IADL scales. The study did not, however, include self-assessment.

With an increasing number of individuals in need of care and limited availability of health and social care professionals, there would be value in having an instrument for self-assessment of social and health functioning. In the study of Brandão et al. (2011), selected parts of the EASYCare questionnaire were used for self-assessment. To the best of our knowledge, to date, the whole EASYCare questionnaire has not been validated for self-assessment.

Our research questions are thus: does self-assessment deliver results as valid as the professional one; our hypothesis therewith is that it does (aspects of construct validity; Terwee et al. 2007), and do results of self-assessment correlate with the ones obtained with reference (gold standard) instruments used in geriatric practice (aspects of concurrent validity that belong to criterion validity as presented in the COSMIN framework; Mokkink et al. 2010)? We therefore compared the results of professional assessment and self-assessment by older participants, and self-assessment against reference instruments in order to determine whether self-assessment has validity for use with some populations of older people who are not frail but at the same time not entirely independent (and not in working age, for whom health risk assessment is recommended; Stuck et al. 2015).

## Method

The project was approved by the bioethical committee of Poznan University of Medical Sciences, Poland.

### Participants

Community-dwelling older people (*n* = 100) entered the study. Participants were volunteers recruited in senior’s centres in the city of Poznan (the fifth largest city in Poland); all persons asked to participate expressed their willingness and were included (response rate of 100%). The inclusion criteria were age (at least 60 years) and absence of severe cognitive impairment [defined as abbreviated mental test score (AMTS) below 7 points]. Participants were provided with a detailed explanation of the study, and an informed consent was obtained. Socio-demographic data were not collected prior to the study, as they are part of the EASYCare questionnaire.

Exclusion criteria were acute conditions or worsening of chronic conditions requiring unplanned medical consultations or a hospitalisation during the observation period (2 weeks). None of the screened persons were excluded.

### Measures

The study was composed of two assessments, done at the homes of the participants, based on the EASYCare Standard 2010 questionnaire, within a period between 1 and 2 weeks. The first assessment was performed by the participants themselves (self-completion), and the second one was completed by EASYCare-trained medical staff members of geriatric team, who are regularly involved in geriatric assessment, including all reference instruments. They were trained in the use of the EASYCare system and were active in its development within its international network. The professionals were blinded for the scores from self-assessment. The results of these two assessments were compared.

During the first assessment, selected tools of CGA were also used to characterise the functional status of each subject. At the beginning, screening of cognitive status with the AMTS was performed. Thereafter, functional status was defined with Barthel Index (Mahoney and Barthel 1965) and Lawton scale (Lawton and Brody 1969); screening for depression with a short form of geriatric depression scale (GDS; Sheikh and Yesavage 1986) was also undertaken.

AMTS is a brief screening tool showing sensitivity and specificity exceeding 80% for dementia with scores ranging from 0 to 11 and a cut-off of <7 points (Jackson et al. 2013). In our study, all subjects had AMTS results over 6 and were included. Barthel Index is a scale for ADL measurement, with lower scores indicating greater dependency. The score value ranges between 0 and 100 points. Lawton scale is an instrument assessing the dependence in IADL, with score values between 0 and 8 points. GDS is a screening tool for self-assessment of the risk of depression. The short version of GDS, composed of 15 questions, was used, delivering results in the range from 0 to 15 points. Subjects with at least 6 points in the GDS scale were classified as having symptoms of depression.

### The EASYCare Standard 2010 questionnaire

The EASYCare questionnaire underwent a complete cultural adaptation in Poland, and its psychometric properties were evaluated (Bień et al. 1999). In the initial part of the questionnaire personal details and medical history were collected. The socio-demographic data included: sex, age, residence area (rural, urban), current marital status (single including separated/divorced and widowed as well as married/cohabiting), formal education (less than primary, primary, vocational, secondary, higher education), living arrangements (alone, with spouse, with extended family), professional status (employed full-time, employed part-time, unemployed, housewife, pensioner, retired, student). Some aspects were formulated as full text questions: “In general how do your family finances work out at the end of the month?” (not enough to make ends meet, just enough to make ends meet, some money left over), “Are you a carer for someone?” (yes/no), “Does someone provide care for you?” (yes/no/other).

The main part of the questionnaire addresses the functioning of an older person in 7 domains by posing simple questions related to their concerns. The first of them, “seeing, hearing and communicating”, includes 4 items, e.g. “Can you use the telephone?”. The second domain, “looking after yourself”, consists of 13 items, e.g. “Can you wash your hands and face?”. The third one, “mobility (getting around)”, has 8 items, e.g. “Can you get around indoors?”. The fourth domain is “safety”, including 5 items, e.g. “Is there anyone who would be able to help you in case of illness or emergency?”. The fifth, “accommodation and finances”, consists of 3 items, e.g. “In general, are you happy with your accommodation?”. The sixth domain, called “staying healthy (prevention)”, has 7 items, e.g. “Has your blood pressure been checked recently?”. The seventh domain, “mental health and well-being”, includes 9 items, e.g. “During the last month, have you often been bothered by feeling down, depressed or hopeless?”.

Three summarising indexes were calculated according to the EASYCare Standard 2010 algorithms, based on the analysis performed within the domains listed above:

- *Independence score*—determines the independence of assessed individual in terms of basic and complex activities of daily living; the final score ranges between 0 and 100 points where higher score indicates greater dependence rate,
- *Risk of breakdown in care*—determines the risk of hospitalisation; final score ranges 0–12 points—higher score defines increased risk of hospitalisation,
- *Risk of falls*—the final score ranges 0–8 points; the scores of 3 or more were classified as increased risk of falls.

*Independence score* contains following items: 1 from the 1st domain (“Can you use the telephone?”), 10 from the 2nd domain (“Can you keep up your personal appearance?”, “Can you dress yourself?”, “Can you use the bath or shower?”, “Can you do your housework?”, “Can you prepare your own meals?”, “Can you feed yourself?”, “Can you take your own medicine?”, “Do you have accidents with your bladder (incontinence of urine)?”, “Do you have accidents with your bowels (incontinence of faeces)?”, “Can you use the toilet (or commode)?”), 6 from the 3rd domain (“Can you move yourself from bed to chair, if they are next to each other?”, “Can you get around indoors?”, “Can you manage stairs?”, “Can you walk outside?”, “Can you go shopping?”, “Do you have any difficulty in getting to public services? (e.g. doctor, pharmacy, dentist, etc.)”), and 1 from the 5th domain (“Are you able to manage your money and financial affairs?”).

*Risk of breakdown in care* consists of following items: 5 from the 2nd domain (“Can you dress yourself?”, “Can you use the bath or shower?”, “Can you feed yourself?”, “Do you have accidents with your bladder (incontinence of urine)?”, “Can you use the toilet (or commode)?”), 1 from the 3rd domain (“Have you had any falls in the last 12 months?”), 1 from the 6th domain (“Do you have any concerns about your weight?”), and 5 from the 7th domain (“In general, would you say your health is: (excellent, very good, good, fair, poor)?”, “Have you had bodily pain in the past month?”, “During the last month, have you often been bothered by feeling down, depressed or hopeless?”, “During the last month, have you often been bothered by having little interest or pleasure in doing things?”, “Do you have any concerns about memory loss or forgetfulness?”).

*Risk of falls* contains following items: 1 from the 1st domain (“Can you see (with glasses, if worn)?”), 4 from the 3rd domain (“Can you move yourself from bed to chair if they are next to each other”, “Do you have any problems with your feet?”, “Have you had any falls in the last 12 months?”, “Can you walk outside”), 2 from the 4th domain (“Do you feel safe inside your home?”, “Do you feel safe outside your home?”), and 1 from the 6th domain (“Do you think you drink too much alcohol?”).

### Statistical analysis

The statistical analysis was performed with STATISTICA 12.0 software (StatSoft, Poland). Descriptive results are presented as means and SD. Numbers of persons with concerns in the individual domains are presented as numbers which equal percentages (*n* = 100). Normality in the data distribution was examined with the Shapiro–Wilk test.

Agreement between patient’s and researcher’s scores (the first research question of our study) on the individual items of the EASYCare Standard 2010 questionnaire was checked using unweighted Cohen’s kappa statistic. For the seven domains of the questionnaire, weighted Cohen’s kappa was used. The kappa statistic is a chance-corrected measure of the inter-rater agreement. Kappa is interpreted as follows: less than 0.40 indicates poor to fair agreement, 0.41–0.60 indicates moderate agreement, 0.6–0.8 represents good agreement, and 0.80–1.00 means excellent agreement (Landis and Koch 1977). Differences between patient’s and researcher’s average score for the EASYCare summarising indexes were calculated with the Wilcoxon signed-rank test.

Correlations between the self-assessments and the results obtained with reference instruments (second research question) were checked with Spearman’s rank correlation coefficient. Dichotomous data were compared with the Chi-square test.

*p* < 0.05 was considered statistically significant.

## Results

### Characteristics of the study sample

The mean age of the participants was 73.6 ± 7.0 years (range: 60–96 years); among them 67 were females (67%).

About 1/5th of the participants were living alone. Less than half were single (43%), while the remaining were married; no one was cohabiting. Most of them (over 60%) had a good financial situation (some money left over from month to month), in spite of the fact that a vast majority (over 90%) were pensioners or retired. All studied parameters, apart from marital status, were comparable in males and females. The marital status differed also in both analysed age cohorts, with more single subjects in the older group (*p* < 0.001). Detailed socio-demographic characteristics are presented in Tables 1 and 2.

**Table 1:** Characteristics of the study sample: socio-demographics

Variable	Total	Group 1 (60–74y) (*n*; %)	Group 2 (over 75y) (*n*; %)
Gender			
Females	67	39 (66.1)	28 (68.3)
Males	33	20 (33.9)	13 (31.7)
Residence area			
Rural	23	14 (23.7)	9 (22.0)
Urban	77	45 (76.3)	32 (78.0)
Marital status			
Single	43	15 (25.4)	28 (68.3)
Married	57	44 (74.6)	13 (31.7)
Living arrangements			
Alone	19	6 (10.2)	13 (31.7)
With spouse	23	17 (28.8)	6 (14.6)
With extended family	58	36 (61.0)	22 (53.7)
Education			
Primary	5	2 (3.4)	3 (7.3)
Vocational	23	16 (27.1)	7 (17.1)
Secondary	41	22 (37.3)	19 (46.3)
Higher education	31	19 (32.2)	12 (29.3)
Financial situation			
Not enough to make ends meet	6	4 (6.8)	2 (4.9)
Just enough to make ends meet	33	21 (35.6)	12 (29.3)
Some money left over	61	34 (57.6)	27 (65.9)
Employment status			
Employed full-time	2	2 (3.4)	0 (0.0)
Employed part-time	3	3 (5.1)	0 (0.0)
Unemployed	0	0 (0.0)	0 (0.0)
Housewife	2	2 (3.4)	0 (0.0)
Pensioner	89	48 (81.4)	41 (100.0)
Retired	4	4 (6.8)	0 (0.0)

**Table 2:** Characteristics of the study sample: reference instrument results

Instrument	Total	Group 1 (60–74y)	Group 2 (over 75y)
AMTS			
Mean ± SD	10.2 ± 1.0	10.3 ± 1.0	10.2 ± 0.9
(median; range)	(10.0; 7–11)	(11.0; 6–11)	(10.0; 7–11)
Barthel			
Mean ± SD	96.3 ± 6.5	96.8 ± 6.9	95.6 ± 5.9
(median; range)	(100.0; 60–100)	(100.0; 60–100)	(100.0; 80–100)
GDS			
Mean ± SD	3.0 ± 2.7	2.9 ± 3.0	3.1 ± 2.3
(median; range)	(2.0; 0–10)	(2.0; 0–9)	(3.0; 0–10)
IADL			
Mean ± SD	6.7 ± 2.0	6.7 ± 2.2	6.8 ± 1.7
(median; range)	(8.0; 0–8)	(8.0; 0–8)	(8.0; 3–8)

Twenty persons reported to take care of someone on a permanent basis. Forty-eight subjects were not independent in IADL and were recipients of informal care.

The participants were relatively independent; no differences in independence were observed between the younger and the older group. In total, 20 subjects had symptoms of depression (GDS result above 5, whereas none had GDS above 10 points).

### Comparison of the two assessments

No significant differences were found in *Independence score* and in *Risk of breakdown in care* between self-assessments and the trained professional’s assessments (8.6 ± 12.0 vs. 9.0 ± 12.7, and 1.0 ± 1.1 vs. 1.2 ± 1.4, respectively). As far as the *Independence score* is concerned, the assessments were slightly different in 26 subjects; in 19 of them, the professional’s scores were higher. The *Risk of breakdown in care* was assessed differently in 8 subjects, where the professional’s score was lower.

Differences in the *Risk of falls* between professional assessment and self-assessment were observed in 21 subjects. In 18 of them, the scores of trained professionals were higher slightly but statistically significantly (1.0 ± 1.1 vs. 1.1 ± 1.4; *p* = 0.005), due solely to the different response to the item “Do you feel safe outside your home?”. There were only 2 subjects in whom the assessment done by trained professional identified increased risk of falls (3 points) whereas self-assessment did not (2 points). In all remaining subjects, the classification of the *Risk of falls* was the same despite score differences.

The difference between two assessments of *Risk of falls* increased with age (*r* = 0.29; *p* = 0.004) and was higher in those who were single in comparison with married individuals (1.2 ± 1.2 and 0.8 ± 1.1; *p* = 0.031). Also, negative correlation was found between the difference and AMTS (*r* = −0.38, *p* < 0.001) and Barthel Index (*r* = −0.45, *p* < 0.001), and positive correlation between the difference and GDS (*r* = 0.20, *p* = 0.043). Moreover, the higher the self-assessment score was the more the trained professional’s score deviated from it (*r* = −0.41; *p* < 0.001).

Excellent to good agreement between self-assessment and professional assessment was found for all 49 individual items of the scale. The Cohen’s kappa values for the seven domains of the EASYCare questionnaire ranged from 0.89 to 0.95; the corresponding data are presented in Table 3.

**Table 3:** Weighted Cohen’s kappa values for the two assessments (self-assessment vs. trained professional assessment) in all domains of the questionnaire

	EASYCare domain	Kappa value
1	Seeing, hearing, and communicating	0.91
2	Looking after yourself	0.95
3	Mobility (getting around)	0.95
4	Safety	0.95
5	Accommodation and finances	0.89
6	Staying healthy (prevention)	0.95
7	Mental health and well-being	0.92

Comparison between both assessments concerning the number of participants with at least one need reported in the individual EASYCare domains showed almost perfectly uniform results (Table 4). All participants reported concerns in at least one of the analysed domains. They demonstrated most concerns in the 6th domain (staying healthy—prevention) followed by the 7th domain (mental health and well-being). The lowest number of concerns was reported in the 1st (seeing, hearing, and communicating), 4th (safety), and 5th (accommodation and finances) domains.

**Table 4:** Participants with at least one concern reported within the EASYCare domains

	EASYCare domain	Self-assessment	Professional assessment
1	Seeing, hearing, and communicating	40	38
2	Looking after yourself	69	69
3	Mobility (getting around)	48	48
4	Safety	39	39
5	Accommodation and finances	42	41
6	Staying healthy (prevention)	99	99
7	Mental health and well-being	89	90

### Self-assessment

The scores of all three summarising indexes were comparable in males and females and were higher in the older age cohort (60–74 vs. 75+): *Independence score* (6.1 ± 11.8 vs. 12.1 ± 11.5, *p* < 0.001), *Risk of breakdown in care* (2.3 ± 2.3 vs. 3.2 ± 2.0, *p* = 0.007), and *Risk of falls* (0.7 ± 1.1 vs. 1.3 ± 1.1, *p* = 0.003). They were also higher in those who were single in comparison with married individuals: *Independence score* (11.3 ± 12.2 vs. 6.5 ± 11.5, *p* = 0.016), *Risk of breakdown in care* (3.1 ± 2.4 vs. 2.4 ± 2.1, *p* = 0.083), and *Risk of falls* (1.2 ± 1.2 vs. 0.8 ± 1.1, *p* = 0.031). A total of 14 persons’ scores indicated increased risk of falls.

All three summarising indexes correlated with the functional status of participants (Table 5). The strongest correlation was observed between the indexes and GDS, the weakest with IADL.

**Table 5:** Correlations between the results of self-assessment with EASYCare Standard 2010 questionnaire and functional status

	Mean ± SD (range)	Versus AMTS	Versus Barthel Index	Versus GDS	Versus IADL
Independence score	8.6 ± 12.0 (0–58)	*r* = −0.30 *p* = 0.002	*r* = −0.51 *p* < 0.001	*r* = 0.41 *p* < 0.001	*r* = −0.26 *p* = 0.010
Risk of breakdown in care	2.7 ± 2.2 (0–11)	*r* = −0.26 *p* = 0.008	*r* = −0.35 *p* < 0.001	*r* = 0.44 *p* < 0.001	*r* = −0.17 *p* = 0.09
Risk of falls	1.0 ± 1.1 (0–5)	*r* = −0.29 *p* = 0.003	*r* = −0.36 *p* < 0.001	*r* = 0.50 *p* < 0.001	*r* = −0.28 *p* = 0.004

*r* Spearman’s rank correlation coefficients, *AMTS* abbreviated mental test score, *GDS* geriatric depression scale, *IADL* instrumental activities of daily living evaluated with Lawton scale

## Discussion

In the past 20 years, the EASYCare system has been developed and spread all over the world. It is a brief standardised method for assessing the functioning of older people based on their concerns. Although there are relatively few studies on the implementation of EASYCare-based interventions in the literature, its ease of use has been widely demonstrated in various settings (Craig et al. 2015). Ritters et al. (2012) presented the data from the EASYCare instrument’s use for self-assessment and found that over 80% of participants reported it as very easy or easy to complete. Complementing these findings, the results of our study point to its usefulness in self-assessment which is a key issue of the personalisation agenda (Abendstern et al. 2014). Notably, the applications of the EASYCare system in self-assessment and in the assessment done by trained professionals have not to our knowledge been compared before.

In our study, the self-assessment scores obtained in the three summarising indexes *(Independence score, Risk of breakdown in care,* and *Risk of falls)* were consistent with the scores obtained using other tools (AMTS, Barthel Index, Lawton scale, GDS), which provides evidence of concurrent validity for self-assessment in the context of functional disability of older people. In our previous study, similar evidence of concurrent validity was found for the trained professional’s perspective in an assessment performed with the EASYCare Standard 2010 questionnaire (Talarska et al. 2016). The strongest correlation of the summarising indexes was observed with the results of GDS screening. This is not surprising as depression is a risk factor of dependence and many other conditions which are common in geriatric care, e.g. malnutrition (Krzyminska-Siemaszko et al. 2016). The weakest correlation was calculated against IADL and Barthel Index which may be partially due to ceiling effects, as the participants had high scores in both scales (Terwee et al. 2007).

We observed excellent agreement between the self-assessment and the assessment performed by trained professionals, and showed the possibility of use of the self-assessment in everyday practice. Our initial hypothesis about construct validity is herewith confirmed. There was a small but statistically significant difference between professional assessment and self-assessment in the *Risk of falls* scores resulting from different response to the question “Do you feel safe outside your home?” It is noteworthy that only in 2 subjects the assessment done by trained professional identified increased risk of falls whereas self-assessment did not.

Ostbye et al. (1997) demonstrated that differences between the researcher’s and patient’s perspectives rise with rising stage of dementia. In our opinion, in the absence of dementia, older individuals can reasonably be expected to complete the EASYCare Standard 2010 questionnaire by themselves or with the help of informal caregivers. They should, however, be informed when to contact a professional caregiver, based on the results of the self-assessment.

As far as the limitations of our study are concerned, it must be stressed that the generalisability of the findings should be restricted to older people with good to moderate functional abilities and those without significant cognitive impairment. We did not include those screened for moderate or higher levels of cognitive impairment; the majority of our respondents were independent in ADL and did not report severe limitations or other problems in their functioning. Our sample included individuals who were better educated than observed in a nationwide representative study of PolSenior (Bledowski et al. 2011) which can be viewed as a limitation for the time being; however, it is worth noting that the proportion of the better educated among older people is rising with time.

For subjects with a higher level of disability, separate studies are necessary. Still, at present it can be stated that for mildly dependent individuals the most important difference between professional assessment and self-assessment was in response to the item “Do you feel safe outside your home?”, subsequently leading to differences for the whole *Risk of falls* index. This seems to indicate that older persons with minor disabilities gave a different interpretation to specific aspects of their functioning outside their homes than the professionals.

The results of our study are complemented by the findings by Ruikes et al. (2016) who used EASYCare TOS (two-step older persons screening instrument) to select frail patients for a multicomponent integrated primary care programme. They found that this instrument had limited effectiveness for the identification of patients in need of integrated care, possibly because they were too frail to benefit from this approach. Thus, early screening with the EASYCare self-assessment may have a place ahead of professional assessment for those who are either not frail or pre-frail, and may help to identify individuals for whom further professional assessment is indicated (Challis et al. 2008). The additional advantage of self-assessment is that it potentially allows to save resources and to free up professional staff to concentrate on the assessment of more complex cases (CSED 2006).
